# Is there scientific relevance to the plot of films and documentaries about eating disorders?

**DOI:** 10.1590/0034-7167-2022-0547

**Published:** 2024-03-15

**Authors:** Alessandra Honorio Boroski, Rosane Pilot Pessa, Janaína Cristina Pasquini de Almeida, Jacqueline de Souza

**Affiliations:** I Universidade de São Paulo. Ribeirão Preto, São Paulo, Brazil

**Keywords:** Anorexia, Feeding and Eating Disorders, Teaching, Audiovisual Media, Films, Anorexia, Trastornos de Alimentación y de la Ingestión de Alimentos, Enseñanza, Medios Audiovisuales, Películas, Anorexia, Transtornos da Alimentação e da Ingestão de Alimentos, Ensino, Mídia Audiovisual, Filmes

## Abstract

**Objectives:**

to analyze films and documentaries about eating disorders from the last twenty years, identifying the way they approach the topic as well as their relevance for didactic use in teaching the health field.

**Methods:**

a descriptive study, whose data collection was carried out on the main streaming and video platforms, resulting in the survey of 60 media. Of these, only 25 had audio/subtitles in Portuguese (inclusion criteria). scientific relevance was analyzed considering psychopathological and epidemiological aspects of these disorders. A questionnaire about the plot, characters and descriptive data analysis were used.

**Results:**

most media were dramas about female teenagers who tried to conform to beauty stereotypes, whose symptoms portrayed converged with current medical diagnostic manuals.

**Conclusions:**

in practical terms, a classificatory list of 11 media was prepared that could be used as a teaching resource for teaching this topic in the health field.

## RESUMO

**Objetivos:**

analisar filmes e documentários sobre transtornos alimentares dos últimos vinte anos, identificando o modo como abordam a temática, bem como sua pertinência para o uso didático no ensino do campo da saúde.

**Métodos:**

estudo descritivo, cuja coleta de dados foi realizada nas principais plataformas de *streaming* e vídeos, resultando no levantamento de 60 mídias. Dessas, apenas 25 tinham áudio/legenda em português (critério de inclusão). A pertinência científica foi analisada considerando aspectos psicopatológicos e epidemiológicos desses transtornos. Utilizaram-se um questionário sobre o enredo, as personagens e análise descritiva dos dados.

**Resultados:**

a maioria das mídias eram dramas sobre adolescentes do sexo feminino que tentavam adequar-se aos estereótipos de beleza, cujos sintomas retratados convergiam com os manuais de diagnósticos médicos atuais.

**Conclusões:**

em termos práticos, elaborou-se uma lista classificatória de 11 mídias que poderão ser utilizadas como recurso didático para o ensino desse tema no campo da saúde.

## RESUMEN

**Objetivos:**

analizar películas y documentales sobre trastornos alimentarios de los últimos veinte años, identificando la forma en que abordan el tema, así como su relevancia para su uso didáctico en la enseñanza del campo de la salud.

**Métodos:**

estudio descriptivo, cuya recolección de datos se realizó en las principales plataformas de *streaming* y video, dando como resultado la encuesta a 60 medios. De estos, sólo 25 tenían audio/subtítulos en portugués (criterios de inclusión). Se analizó la relevancia científica considerando aspectos psicopatológicos y epidemiológicos de estos trastornos. Se utilizó un cuestionario sobre la trama, los personajes y el análisis de datos descriptivos.

**Resultados:**

la mayoría de los medios fueron dramas sobre mujeres adolescentes que intentaban ajustarse a estereotipos de belleza, cuyos síntomas retratados convergían con los manuales de diagnóstico médico vigentes.

**Conclusiones:**

en términos prácticos, se elaboró un listado clasificatorio de 11 medios que podrían ser utilizados como recurso didáctico para la enseñanza de este tema en el campo de la salud.

## INTRODUCTION

Eating disorders (ED), described as persistent disorders related to eating patterns, are characterized by behaviors that culminate in extreme weight loss, obesity, or other physical and emotional problems with negative impacts on individuals’ health and psychosocial functioning, in addition to the sociocultural context that disseminates certain stereotypes related to beauty standards in society^(1-5)^.

In contemporary Western culture, the ideal of female beauty is mostly associated with thinness. This biotype has also been seen as a symbol of greater competence, more success, self-control and social attractiveness, corroborating the notion of body-instrumentality, i.e., the idea that physical “improvement” body through diets and exercises would culminate in greater emotional control and social achievement^(6-7)^.

The internalization of socially promoted beauty ideals has been an issue addressed by most studies on ED^(5-12)^, whose authors point out both the consequent concerns with body image resulting from ideals that are often unattainable and the role of sociocultural influences in the internalization of the ideal of thinness, especially among women, emphasizing the media’s strong contribution in this sense.

It is argued that, in general, the bodies shown in the media are images that do not represent the human body’s cultural and natural expressions. Known as “body-media”, they are not equivalent to the natural body, but they are one of the main resources used for advertising campaigns for different products^(2,7,13)^.

In this regard, several authors have highlighted the role of the media in producing and maintaining ideologies, reinforcing or combating certain stereotypes, representing popular discourse and forming a collective culture related to certain phenomena^(1,7,12,14)^. As a result, the demands imposed by fashion and the media on body image have been identified by some studies as factors that have a negative impact, especially on women’s and young people’s mental health^(1,6)^.

Previous studies on media and ET aimed to analyze the impacts caused by the media through the production of aesthetic and dietary beauty standards in different environments and stages of life^(2,4-6,8-11,13-20)^. In general, such studies assessed the perception of body self-image, eating behavior and nutritional status, and some highlighted the influence of social networks in relation to individuals’ body image^(9,18,21)^.

Specifically at the international level, the focus of both field^(5-6,20,22)^ and review^(14,21,23)^ studies on media influence has been referring to the internalization of beauty standards, especially by the female population, resulting Western culture and the search for physical standards also imposed by society. On the national scene, recent literature that addresses the topic deals with the media’s influential power on the younger population, taking into account the lack of preparation, in educational terms, of this public when using social networks as well as unique aspects inherent to the adolescence phase, such as the search for acceptance by peers and insecurity related to physical appearance, configuring them as a group of risk for developing ED^(9,15-16,24)^.

It is noteworthy that, of these studies, only one compiled evidence on television media, specifically advertisements and their influence on children and young people^(13)^. The others referred to printed media (women’s magazines)^(19)^, social networks^(2,18,20-21)^ and blogs, or to the media in general, without specifying the modality^(4-5,8-9,14-17,22)^. This overview highlights the innovative potential of using cinematographic media as a source of data for research on the topic.

Furthermore, it is worth highlighting that an important review study developed in Europe^(14)^ highlights that, despite the large number of research on the dissemination of unrealizable ideals of beauty by the media and their consequences on the development of ED, There is a lack of studies on the consequences and role of the media in the progression and coping by people who already have these disorders. Thus, these authors highlight the importance of developing studies that analyze how these disorders are addressed by different types of media, in order to deepen the understanding of how such representations also influence the group of people who are already ill.

The present study, therefore, aims to contribute to filling this gap, as it focuses on cinematographic media, emphasizing the approach to ED and discussing their possible influences on the development and course of such a disease as well as the scientific relevance of such representations and the possibility of didactic use of some of these productions.

## OBJECTIVES

To analyze films and documentaries about ED from the last twenty years, identifying how they approach the topic as well as their relevance for didactic use in teaching the health field.

## METHOD

### Ethical aspects

In the present study, public domain information available on freely accessible platforms was used. Therefore, the study falls within the sole paragraph of Article 1 of Resolution 510/2016 of the Brazilian National Health Council, which determines specific ethical guidelines for the human and social sciences and does not require assessment by the Research Ethics Committee/National Commission of Research Ethics (REC/CONEP (*Comissão Nacional de Ética em Pesquisa*)).

### Study design, period and place

This is an exploratory descriptive study, which was developed from August 2019 to July 2020 based on data obtained from cinematographic productions, such as films and documentaries produced in the last 20 years, counting from the period in which the study was carried out. The method was structured based on a study adaptation on documentary analysis of written media proposed by Souza *et al*. (2020)^(25)^ and also the items described in the Meta-analyses Of Observational Studies in Epidemiology (MOOSE).

### Procedures for sample composition

The survey of productions to be analyzed was carried out in two stages. The first stage consisted of defining the set of media produced in the period listed (“study population”). The second stage consisted of identifying the media that met the eligibility criteria for the analysis itself (“sample”).

In the first stage, the descriptors were used, in Portuguese and English, “eating disorder”, “media”, “anorexia”, “bulimia”. The platforms used were the Internet Movie Database (IMDB), which allows media consultation, Netflix, a global provider of films and television series via streaming, Globoplay, a digital on-demand video streaming platform created and developed by *Grupo Globo*, and Google Play, with a digital distribution service for applications, games, films, television programs, music and books, developed and operated by Google. This search resulted in a list of 100 titles, of which 40 were repeated and 35 did not have audio or subtitles in Portuguese (inclusion criteria), resulting in a sample of 25 titles, 16 films and nine documentaries.

### Data collection

Data were collected by two study authors trained for this purpose and with expertise in the topic in question. A questionnaire previously prepared by the research group was used and assessed by two external researchers, such as a nutritionist with expertise in teaching and researching such disorders and a nurse with a doctoral degree in psychiatric nursing with care experience in primary care units.

**Figure 1:**
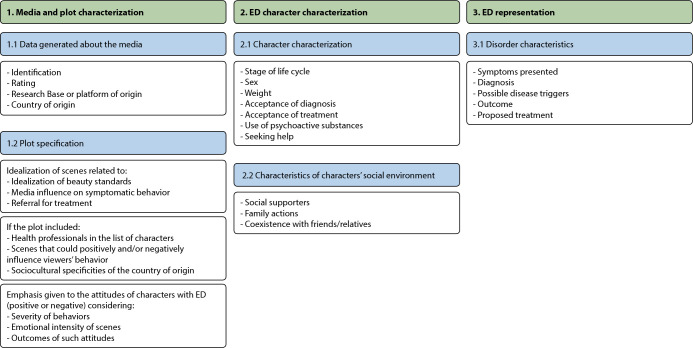
Structure and detail of questionnaire items used in data collection, Ribeirão Preto, São Pulo, Brazil, 2020

Figure 1 shows the structure and items of the questionnaire used. As can be seen, this was structured into three major topics: 1) media and plots; 2) characters with ED; and 3) ED. These topics encompassed five guiding items, namely: 1.1) general media data; 1.2) plot specifics; 2.1) character characteristics; 2.2) characteristics of their social environment; and 3.1) disorder characterization. Furthermore, the instrument included the minutes of the scenes that characterized the analyzed items.

All 25 cinematographic media were watched in full with concomitant completion of the questionnaire by the two authors of the study from February to March 2020. Doubts and inconsistencies in filling out the items were resolved with the study supervisor considering the corresponding scenes and minutes.

### Data analysis

The analysis was undertaken in three stages. The first aimed to provide a general overview of the media listed. Firstly, the percentage of plots that corresponded to films or documentaries were identified as well as the number of media classified in each genre (drama, comedy, romance and musical). Then, their distribution was broken down according to the year of production. We also analyzed descriptively (n and %) distribution of media according to sex, age group and availability of social support from the main characters.

In the second stage of analysis, the purpose was to describe the way in which ED were approached. In this item, the data from each media were analyzed separately to discriminate which of them presented scenes that referenced the main character’s weight, illustrated the dissemination of beauty stereotypes, showed the characters’ attempts to conform to such stereotypes, represented the main character’s relationship with family members and different types of clinical outcomes. Then, the respective scenes highlighted in the minutes were described in detail, in order to obtain concrete examples of the characteristics of such scenes.

Still at this stage, we analyzed how many symptoms of ED were represented in each media and their correspondence with the Diagnostic and Statistical Manual of Mental Disorders, 5^th^ edition (DSM-5)^(3)^. The number of media that represented each disorder (anorexia or bulimia) and respective triggering factors were analyzed, classifying them according to media appeal and/or family stress. Disorder triggers, diagnoses, proposed treatments and characteristics of the social environment represented in each of the plots were compared with the literature related to the psychopathological and epidemiological aspects of this condition.

Analyzes of each plot were also undertaken to list the number of media that illustrated behaviors with the potential to be reproduced by viewers, and the respective scenes were also descriptively detailed. These descriptions were then categorized into the following topics: refusal to seek treatment or ask for help; harmful diets; ways to induce vomiting; exaggerated exercise routine. It was then listed how many and which media represented each of these categories.

The third stage, therefore, corresponded to the classification of titles considered relevant in scientific terms and which, therefore, would be appropriate for use for teaching purposes. To this end, it was considered as a criterion that the media presented at least three of the five aspects in the plot: 1) health professionals’ work; 2) some aspect related to diagnosis; 3) referral for treatment; 4) greater number of symptoms of the disorder; and 5) plots with greater emphasis on characters’ positive attitudes towards the disorder.

Thus, the media that met this criterion were listed (n=11). It is noteworthy that, of these, five met all the criteria and were referred to a third analyst with expertise in teaching such disorders, who watched the selected media in full, also focusing on the characteristics emphasized in the indicated minutes, in order to provide greater quality control of interpretations and validate such indication.

Regarding the assessment of the relevance of adopted criteria and the media listed in this final list, Fisher’s exact test was carried out to analyze the association between “media considered to disseminate information based on scientific knowledge” (yes/no) and the following variables: “presence of health professionals in the plot” (yes/no); “the main character is referred for treatment” (yes/no); “number of symptoms that corroborate the DSM-5 criteria” (up to three/four or more); “plots with an emphasis on characters’ positive attitudes towards the disorder” (yes/no); “outcomes for the character in the plot” (condition improvement/worsening or maintenance). The purpose was to validate the significance of these items in the composition of the criteria used to consider relevant media in scientific terms (significance level p<0.05).

## RESULTS

### Media general overview

**Figure 2:**
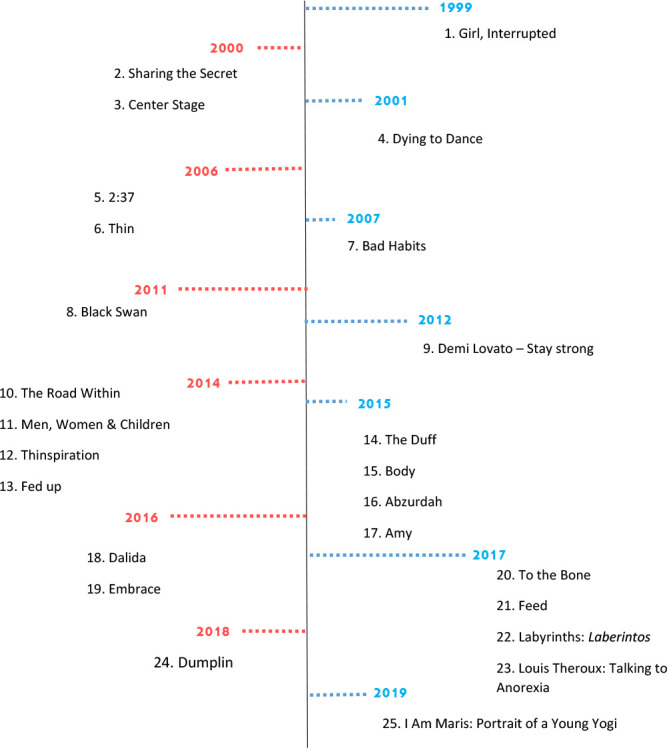
Distribution of media analyzed from the questionnaire, Ribeirão Preto, São Paulo, Brazil, 2020 (N=25)

The media analyzed were ordered according to a timeline. Thus, 16 (64%) were films and nine (36%) were documentaries, the majority (64%) being produced in the United States with an emphasis on the years 2014, 2015, 2017 and 2018 (Figure 2).

It is worth noting that approximately half of these media (48%) did not correspond to a single cinematographic genre. Therefore, the predominant genre was drama (n=16), and four of the media in this genre were biographical films or documentaries. The other genres identified were, respectively, comedy (n=5), romance and musical (n=3), and always linked to some other genre.

In relation to clinical outcomes, according to Table 1, the majority of characters with ED showed improvement (evidenced by their self-report or scenes of them resuming functional eating habits, interacting with the support network, accepting help and undergoing treatment) or the clinical picture remained unchanged.

**Table 1:** Distribution of media according to the characteristics of the main characters with eating disorders, Ribeirão Preto, São Paulo, Brazil, 2020 (N=25)

Variables	n	Corresponding media^*^
Main characters with eating disorder		
Sex		
Female	24	1-12, 14-25
Male	1	13
Age group		
Child	1	13
Adolescent	17	1-5, 9-12, 14, 16, 20-25
Adult	7	6-8, 15, 17-19
Receives social support	21	1-4, 6, 9-10, 12-25
Highlight scenes related to the topic		
Reference to the main character’s weight	6	4, 6-7, 12, 16, 18
Attempt to adapt to beauty standards^†^	23	1-16, 18-24
Broadcasting a beauty stereotype^†^	20	1-2, 4, 6-7, 9-14, 16-20, 22-25
Television	11	1-2, 7, 9-10, 13, 17-19, 23-24
Internet in general	12	9-13, 16-17, 19-20, 22-23, 25
Social networks	8	9, 14, 17, 19-20, 23-25
Main reaction of family members^†^		
Indifference	11	3, 7-8, 10-11, 14-18, 20
Treatment support	13	1-2, 4, 6, 9, 12-13, 16, 20-23, 25
Clinical outcomes		
Worsening^‡^	5	7-8, 10, 17-18
Improvement	8	2, 9, 12, 16, 20-22, 25
Unchanged	8	1, 3-6, 11, 15, 23

* *The number of media corresponds to the numbering in Figure 1;*

† *As these items can be considered subjective and subject to interpretation bias, in the following paragraphs, scenes are presented that exemplify the content of the analysis undertaken;*

‡ *Death (n=3), suicide (n=1) and attempted suicide (n=1).*

### How to approach the disorder

In most media, the main characters with ED were female adolescents. The standout scenes related to the topic were those in which the character with ED tried to adapt to some beauty standard and others that showed media coverage of a beauty stereotype (Table 1).

In the film “Center Stage”, at 15’, the main character, at a party, observes how other women dress and act, then she goes to the bathroom and gets ready in a similar way to what she observed in other women (cleavage, loose hair, makeup). In the film “The minimum to live”, at 3’, the main character does exercises looking at the drawings she made with thin bodies and protruding bones fixed to the wall. These are some examples of scenes in which the main character tried to conform to beauty standards.

Regarding the dissemination of a beauty stereotype, some examples are in the film “Thinspiration”, at 8’, in which the main character’s friend shows her a website called “thininspiration”, which promotes diets and photos of women thin and with prominent bones, and in the film “Dying to Dance”, at 19’, in which the main character weighs herself, writes down her weight on paper and sticks it on her mirror next to magazine clippings with thin women’s bodies and diet tips.

Regarding the family’s reaction, an example of indifference can be seen in the film “DUFF”, at 41’, in which the main character is telling her mother what she felt about a boy and how she needed to change for him to pay attention to her; his mother interrupted him and went back to talking about herself. On the other hand, “Sharing the Secret”, at 71’, illustrates the family supporting treatment. The main character’s mother was in the bathroom with her, saying she loved her and did not want to see her hurt, and then the adolescent vomited, they both cried and the main character asked her mother for help. In the later scene (71’), her mother visited the main character, who was hospitalized for treatment, taking clothes and talking to her in an understanding way.

In all the plots analyzed, at least one symptom of ED consistent with the DSM-5^(3)^ was presented, and, in a large part of them (n=11), people with the disorder presented four or more symptoms with this characteristic. It is noteworthy that in almost half of the plots (n=12) the main character received a formal diagnosis of ED and, in all these cases, the manifestations she presented corresponded to the symptoms described in DSM-5^(3)^ and corroborated the most recent epidemiological data on this disorder. Among the diagnoses presented by the characters, anorexia was the most prevalent (n=10).

Regarding the factors that possibly contributed to the development of characters’ ED in the plot, media appeal was the most prevalent factor (n=18), followed by family stress (n=11). In all plots, there are scenes that illustrate behaviors that have the potential to be reproduced by viewers and, in most of them (n=13), there is greater emphasis on characters’ negative attitudes, such as refusal to seek treatment or ask for help (media 1, 8, 10-14, 16, 18, 21 and 23), harmful diets (1, 7, 10-14, 16, 18, 21 and 23), ways to induce vomiting (5, 8, 10, 12, 16 and 18) and exercise routine that exceeds individual physical capacity (8, 12-13, 21 e 23).

### Scientific relevance in didactic terms for the health field

In films and documentaries that met the criteria for disseminating information based on scientific knowledge (n=11), the plots, in general, emphasized the positive attitudes of characters with ED, and the main characters presented more than three symptoms consistent with the DSM-53 and received treatment in a health unit (Table 2). The main characteristics that resulted in the exclusion of 14 media was the presentation of a plot with emphasis only on the stereotype of the biotype and behavior of people with ED, without a clear explanation of the complexity of the development of such disorders.

**Table 2:** Distribution of films and documentaries that met the criteria related to dissemination of information based on scientific knowledge according to the specificities analyzed, Ribeirão Preto, São Paulo, Brazil, 2020 (n=11)

Characteristics	Scientific knowledge (n=11)		
	n	%	*p* value
Greater emphasis on characters’ positive attitudes^*^	8	73	0.047
Presence of health professionals in the plot	10	91	0.122
Symptoms based on DSM-5			0.017
3 or less	3	27	
4 or more	8	73	
Main character receives treatment	11	100	0.001

* *As this item can also be considered subjective and subject to interpretation bias, in the following paragraph, scenes are presented that exemplify the content of the analysis undertaken.*

In the documentary “Thin”, at 10’30”, the group therapist asks someone to present the group’s rules and objective; several patients are present and one states that the group is a space to ask for or receive support. At 25’50”, a therapist assists a young woman accompanied by her father, admitted to the institution. Both describe how they feel about their daughter’s suffering situation and are welcomed by the therapist who provides a space for the reports. These scenes illustrate one of the media in which the plot placed greater emphasis on characters’ positive attitudes. Another example of this can be seen at 68’ of the film “Thinspiration”, in which the main character creates a website called “beautiful is my body”, which propagates messages of body self-acceptance.

Table 3 presents the titles of films and documentaries that met the criteria related to dissemination of information based on scientific knowledge, detailing the most relevant characteristics and highlighting the five media considered most relevant for the psychopathological and care teaching of ED.

**Table 3:** Title of the media that met the criteria related to dissemination of information based on scientific knowledge (n=11) and those most relevant for teaching purposes (n=5; the first five on the list), Ribeirão Preto, São Paulo, Brazil, 2020

Titles	Diagnosis	Emphasis on positive attitudes	Health professionals	More DSM-5 symptoms	Treatment
To the Bone	*√*	*√*	*√*	*√*	*√*
Louis Theroux - Talking to Anorexia	*√*	*√*	*√*	*√*	*√*
Labyrinths: Laberintos	*√*	*√*	*√*	*√*	*√*
Thin	*√*	*√*	*√*	*√*	*√*
I Am Maris: Portrait of a Young Yogi	*√*	*√*	*√*	*√*	*√*
Body	*X*	*√*	*√*	*√*	*√*
Amy	*X*	*√*	*√*	*√*	*√*
*Sharing the secret*	*X*	*√*	*√*	*√*	*√*
*Dying to Dance*	*X*	*√*	*√*	*√*	*√*
*Stay Strong*	*X*	*√*	*X*	*√*	*√*
The Road Within	*X*	*X*	*√*	*√*	*√*

## DISCUSSION

As for how the topic is approached, in most plots, some sign, symptom or diagnosis is presented as well as a characterization of main characters compatible with scientific literature. There is an emphasis on anorexia nervosa and the repercussions of media appeal and family stress on the development and course of ED.

Despite this scenario, when adopting criteria related to scientific relevance, it was identified that a large part of the media emphasized stereotypes related to a woman’s body type instead of portraying the complexity of the disorder. Regarding the indication for use for teaching purposes, what differentiated the five most recommended media was the presentation of scenes that reliably portrayed ED diagnosis as well as the emphasis on the complexity of the development, course and outcome of this phenomenon.

The idea of “body worship” is marked by the end of the 20^th^ century and the beginning of the 21^st^ century, a period with a strong emphasis on the obsessive search for the perfect body. This search, especially for women from the urban middle classes, became a lifestyle^(26)^. In this sense, the increasing and greater broadcasting of films and documentaries about ED in recent years, as pointed out by the results of this study, suggests the influence of this idea in relation to people’s interest in the topic and possible increase in audience for media that discuss the same.

The way in which characters with ED were represented in the plots, for the most part, corresponded to the current diagnostic criteria as well as the main epidemiological data related to the most prevalent characteristics in people with this disorder (mostly female adolescents and young adults)^(9,15,20)^. It was also identified that the majority of these plots were produced in the United States, which certainly promotes the dissemination of specific aspects of that country (biotype, clothing and routine), potentially exerting, implicitly, a certain influence on viewers from other cultures, corroborating previous studies that deal with the influence of globalization and westernization phenomena on the development of ED^(6,23)^.

Thus, it is worth highlighting that the sociocultural reflection on the development and course of ED has been widely discussed in the literature, which highlights the potential influence of cultural patterns from developed and Western countries on others and the weight of globalization in the dissemination of such patterns^(6,23,27)^. A review study argues that the phenomenon of “westernization”, in itself, does not explain the increase in rates of such disorders in different countries, pointing out that industrialization and urbanization have been factors enabling change in different spheres of society. The authors of this review understand that such changes have revealed previously unknown manifestations in different cultures, and this has been reflected in the discrepancy in ED prevalence rates among countries. They therefore corroborate the notion that multiple factors must be considered in the discussion of such a phenomenon and that sociocultural specificities have important weight, although they are not yet as elucidated in scientific studies^(27)^.

It was identified that the media emphasized the possible media influence on people in relation to their self-image and the development of ED. Thus, another study describes that talking about this topic in itself constitutes a risk factor for the development of anorexia and bulimia in susceptible groups^(28)^.

Additionally, it is worth highlighting that the main characteristics that differentiate cinematographic media from others are the time and intensity of viewers’ exposure to the plot and the idea conveyed, further reinforcing its possible influential character. Thus, the results presented emphasize a certain dichotomy in relation to such media, because, while they provide entertainment, they are also a powerful influencer of behavior and may constitute an additional risk factor, especially for the most emotionally vulnerable groups.

Therefore, in terms of prevention, it is understood that it would be essential for media with these characteristics to follow content warning (trigger warnings), in order to provide support to viewers for making decisions about access to it as well as indicate possible resources for those who identify themselves or need support in this regard, such as the warning provided by Netflix in relation to the series “13 Reasons Why” (“content warning: this series contains scenes that viewers may find disturbing, including graphic images of sexual assault, drug use and suicide. If you or someone you know is facing a difficult time and needs help, visit 13ReasonsWhy.info for more information”)^(29)^. Furthermore, the resurgence of public policies and strategies related to the promotion of mental health with a focus on strengthening the psychological resources of vulnerable groups and their support networks must be prioritized despite any censorship or control mechanism.

Despite all these reservations, it is considered that the didactic use of cinematographic media, as long as they are carefully selected, encompasses numerous dimensions, such as pedagogical, ethical and psychosocial. Thus, the use of films/documentaries constitutes a powerful resource that encourages critical thinking, promoting reflection, observation and encouraging debates, thus being able to expand conceptual knowledge and promote dynamic teaching on the subject^(1)^. In terms of didactic purposes, most of the cinematographic media analyzed were considered relevant for use in psychopathological and care teaching for students in the health area according to the criteria used in the present study.

### Study limitations

The data collection process, however objective it may have been, implied, in a certain way, the author’s interpretation of the scenes watched and, therefore, was subject to some bias. However, in order to minimize it, the validation strategy was used by a third analyst, recording the minutes of the scenes for discussion with group members, explaining the details of the content analyzed in each of the media so that readers can also problematize their perceptions about the propositions listed here. Furthermore, statistical tests were carried out to validate the criteria adopted for selecting the most pertinent plots in scientific and cultural terms, considering the different variables used.

### Contributions to nursing, health, or public policy

In practical terms, the classification list of films and documentaries provides useful information to support nursing teaching in the mental health field, considering that the careful selection of these media covered the psychopathological, psychosocial and ethical dimensions in the development of ED. In that regard, the present study contributes in an important way by investigating and making available this widely accessible media selection that can also be used as a teaching resource in health professional training improvement.

## CONCLUSIONS

ED, in most plots, were represented in a manner compatible with social reality and scientific literature on the subject. Based on the criteria adopted, 11 of the 25 media were considered capable of being used for teaching purposes in health courses. Of these, two films and three documentaries stood out for presenting the most relevant characteristics and scenes with regard to the reliability of symptoms and diagnosis as well as for raising important reflections on the repercussions of this disease on the rehabilitation process’ social environment and challenges. It is noteworthy that indications are permeated by descriptive examples of scenes related to family support, the emphasis on characters’ positive attitudes and their search for help, facilitating the choice and justifying its potential as a didactic resource in health professional training.
