# ERRATUM

**DOI:** 10.1590/0034-7167.20247702e06

**Published:** 2024-06-14

In the article “Is there scientific relevance to the plot of films and documentaries about eating disorders?”, with DOI number: https://doi.org/10.1590/0034-7167-2022-0547, published in Revista Brasileira de Enfermagem, 2024;77(1):e20220547, page 7:

Where it read:

**CONTRIBUTIONS**

Boroski AH, Pessa RP and Almeida JCP contributed to the conception or design of the study/ research. Boroski AH, Pessa RP and Almeida JCP contributed to the analysis and/or interpretation of data. Boroski AH, Pessa RP and Almeida JCP contributed to the final review with critical and intellectual participation in the manuscript.

Read:

**CONTRIBUTIONS**

Boroski AH, Pessa R P, Almeida JCP and Souza J contributed to the conception or design of the study/research. Boroski AH, Pessa RP, Almeida JCP and Souza J contributed to the analysis and/or interpretation of data. Boroski AH, Pessa R P, Almeida JCP and Souza J contributed to the final review with critical and intellectual participation in the manuscript.

