# Supplementary material for: ERRATUM
Source: Rev Bras Enferm. 2024 Jun 14;77(2):e2024n2e06. doi: 10.1590/0034-7167.20247702e06 (PMC11178307; doi:10.1590/0034-7167.20247702e06)
Supplement: Related texts

# Correction: ERRATA

**DOI:** 10.1590/0034-7167.20247702e06pt

No artigo “Há pertinência científica no enredo dos filmes e documentários sobre transtornos alimentares?”, com número DOI: https://doi.org/10.1590/0034-7167-2022-0547pt, publicado no periódico Revista Brasileira de Enfermagem, 2024;77(1):e20220547, página 7:

Onde se lia:

**CONTRIBUIÇÕES**

Boroski AH, Pessa RP e Almeida JCP contribuíram com a concepção ou desenho do estudo/ pesquisa. Boroski AH, Pessa RP e Almeida JCP contribuíram com a análise e/ou interpretação dos dados. Boroski AH, Pessa RP e Almeida JCP contribuíram com a revisão final com participação crítica e intelectual no manuscrito.

Lê-se:

**CONTRIBUIÇÕES**

Boroski AH, Pessa RP, Almeida JCP e Souza J contribuíram com a concepção ou desenho do estudo/pesquisa. Boroski AH, Pessa RP, Almeida JCP e Souza J contribuíram com a análise e/ou interpretação dos dados. Boroski AH, Pessa RP, Almeida JCP e Souza J contribuíram com a revisão final com participação crítica e intelectual no manuscrito.
